# Proapoptotic Bcl-2 inhibitor as host directed therapy for pulmonary tuberculosis

**DOI:** 10.21203/rs.3.rs-4926508/v1

**Published:** 2024-09-02

**Authors:** Sanjay Jain, Medha Singh, Mona Sarhan, Nerketa Damiba, Alok Singh, Andres Villabona-Rueda, Oscar Nino Meza, Xueyi Chen, Alvaro Ordonez, Franco D’Alessio, Eric Aboagye, Laurence Carroll

**Affiliations:** Johns Hopkins University School of Medicine; Johns Hopkins University School of Medicine; Johns Hopkins University School of Medicine; Johns Hopkins University School of Medicine; Johns Hopkins University School of Medicine; Johns Hopkins University School of Medicine; Johns Hopkins University School of Medicine; Johns Hopkins University School of Medicine; University of Pennsylvania; Johns Hopkins University School of Medicine; Imperial College, London; Johns Hopkins University School of Medicine

## Abstract

*Mycobacterium tuberculosis* establishes within host cells by inducing anti-apoptotic Bcl-2 family proteins, triggering necrosis, inflammation, and fibrosis. Here, we demonstrate that navitoclax, an orally bioavailable, small-molecule Bcl-2 inhibitor, significantly improves pulmonary tuberculosis (TB) treatments as a host-directed therapy. Addition of navitoclax to standard TB treatments at human equipotent dosing in mouse models of TB, inhibits Bcl-2 expression, leading to improved bacterial clearance, reduced tissue damage / fibrosis and decreased extrapulmonary bacterial dissemination. Using immunohistochemistry and flow cytometry, we show that navitoclax induces apoptosis in several immune cells, including CD68 + and CD11b + cells. Finally, positron emission tomography (PET) in *live* animals using novel, clinically translatable biomarkers for apoptosis (^18^F-ICMT-11) and fibrosis (^18^F-FAPI-74) demonstrates that navitoclax significantly increases apoptosis and reduces fibrosis in pulmonary tissues, which are confirmed using post-mortem studies. Our studies suggest that proapoptotic drugs such as navitoclax can improve pulmonary TB treatments, and should be evaluated in clinical trials.

## INTRODUCTION

Despite being preventable and treatable, tuberculosis (TB) remains the second leading cause of mortality globally, with an estimated 1.3 million deaths and 10.6 million new cases due to TB, reported in 2022^1^. The number of new TB cases represents the highest incidence recorded since the World Health Organization (WHO) initiated global TB surveillance in 1995, surpassing the pre-pandemic baseline observed in 2019. Further, the burden of drug-resistant TB [DR-TB, including multidrug resistant (MDR)-TB strains resistant to first-line TB drugs rifampin and isoniazid] also increased and there were an estimated 410,000 new cases of rifampin resistant or MDR TB in 2022. The global community has established a goal to eradicate the TB epidemic by 2030, but achieving this objective necessitates urgent and innovative treatments.

During TB infection, early-stage apoptosis is host protective which results in immune clearance of *Mycobacterium tuberculosis-infected* cells, activating both innate and adaptive immune response^2,3^. At later stages, bacteria benefit from inhibiting apoptosis and promoting uncontrolled necrosis, which facilitates infection dissemination and disease persistence^4–6^. Necrosis increases TB-associated morbidity as it causes tissue destruction, promotes fibrosis, and thereby reduces the penetration of antibiotics to the regions where they are needed most. The necrotic granuloma also provides a breeding ground for *M. tuberculosis* replication and can transform into cavities, leading to an increased likelihood of developing drug resistance, failing treatment, and disease transmission^7^. Necrotic tissues, often heal by fibrosis, leading to lung dysfunction long after treatment completion, which is increasing being recognized as post-TB lung disease^8^. Conversely, apoptosis is host-protective by eliminating infected cells without triggering excessive inflammation^9–12^. Therefore, there has been recent interest in developing host-directed therapies (HDTs) that promote apoptosis^13^, and which could shorten the duration of TB treatments when given in combination with antibiotic regimens. Unlike antibiotics, HDTs modulate host cell responses to improve overall outcomes^14–15^, and currently, there are no clinically approved HDTs for pulmonary TB. Importantly, since HDTs target host (mammalian) cells without direct antibiotic effects, they are likely to work against drug-susceptible, as well as MDR *M. tuberculosis* strains^16^.

Here, we study navitoclax (ABT-263), an orally bioavailable, proapoptotic small molecule Bcl-2 inhibitor in clinical trials for cancer treatments, as an adjunctive HDT for pulmonary TB. Addition of navitoclax to the first-line, standard TB treatment (rifampin – R, isoniazid – H and pyrazinamide – Z, RHZ regimen) promotes pulmonary bacterial clearance and reduces lung damage in mouse models of TB, by inhibiting tissue Bcl-2 expression. Further, positron emission tomography (PET) in *live* animals with ^18^F-ICMT-11, a clinically translatable imaging biomarker for apoptosis that targets activated caspase 3/7, demonstrated higher tissue apoptosis in navitoclax-treated animals, which was confirmed using post-mortem analysis (Bcl-2, Bid, Annexin V, caspase 3). Using immunohistochemistry and flow cytometry, we also demonstrate that navitoclax induces apoptosis in multiple cell types, including CD68 + immune cells. Finally, addition of navitoclax to the standard TB treatment significantly reduces pulmonary fibrosis in *live* animals, as measured by ^18^F-FAPI-74 PET, a clinically translatable imaging biomarker for fibrosis (**Fig. S1**), and confirmed on post-mortem analysis (soluble collagen levels and Masson’s trichrome stains). Extra-pulmonary bacterial dissemination was also decreased in animals receiving adjunctive navitoclax.

## RESULTS

### Co-administration of Rifampin does not affect Navitoclax levels in mice

Studies have shown that co-administration of navitoclax with rifampin moderately decreases (40%) navitoclax plasma levels in patients but does not change the C_max_, half-life or its safety profile^17^. We measured navitoclax levels using mass spectrometry in *M*. *tuberculosis-infected* mice co-administrated with rifampin as part of the standard TB treatment. The median (interquartile range) for navitoclax plasma and lung levels were 28.50 (25.65–28.75) μg/mL and 5.76 (5.38–11.32) μg/g, respectively (**Fig. S2**), and consistent with published navitoclax levels achieved in mice without co-administration of rifampin^18,19^.

### Navitoclax administration has no effect on platelet counts in mice

Given that reversible thrombocytopenia is the only major side effect of navitoclax in human studies^20^, we measured the platelets in blood samples from *M*. *tuberculosis*-infected mice (**Fig. S3**). The median platelet counts in untreated mice, and those receiving standard TB treatments, with and without navitoclax were 1.09 × 10^6^ / μL, 1.05 × 10^6^ / μL and 0.99 × 10^6^ / μL, respectively. There were no significant differences in the median platelet counts in mice receiving standard TB treatments with and without navitoclax (*P* = 0.43).

### Navitoclax reduces Bacterial burden and Lung pathology

**Figure 1:**
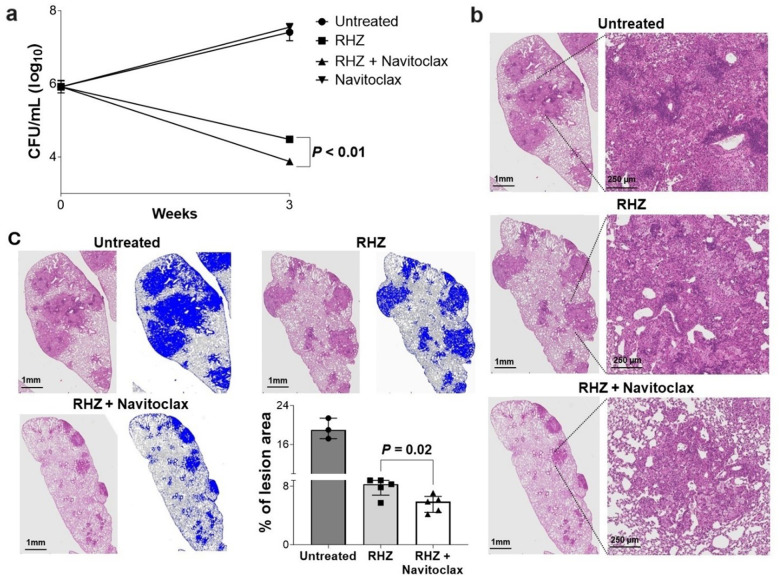
Navitoclax treatment in mouse model of pulmonary tuberculosis at human equipotent dosing. *M*. *tuberculosis*-infected mice were randomly allocated to receive standard TB treatment (R, rifampin; H, isoniazid; Z, pyrazinamide) with or without navitoclax at human equipotent dosing via oral gavage. **a,** Bacterial burden [colony-forming unit (CFU) per mL (log_10_) from whole lung] after three weeks of treatment (n = 4 mice/regimen). **b,** Hematoxylin & Eosin (H&E) stained lung sections of mice demonstrating lung pathology. **c,** H&E-stained lung tissue sections were used to quantify the percentage of affected lung tissue regions. For CFU, data are represented as mean ± standard deviation and statistical comparisons were made using the student t-test. For affected lung tissue regions, data are represented as median ± interquartile range. Statistical comparisons were made using the Mann-Whitney U test.

TB treatments were initiated three weeks after an aerosol infection with *M. tuberculosis*. While treatment with navitoclax alone did not have any antimicrobial effects, when combined with the standard TB treatment (RHZ + navitoclax), there was a significant reduction in the bacterial burden compared to the standard treatment alone (RHZ) (*P* < 0.01) (Fig. 1a). Addition of navitoclax also improved lung pathology (Fig. 1b), with a significant decrease in the percentage of affected lung regions (Fig. 1c).

### Navitoclax induces Lung tissue apoptosis by inhibiting Bcl-2

**Figure 2:**
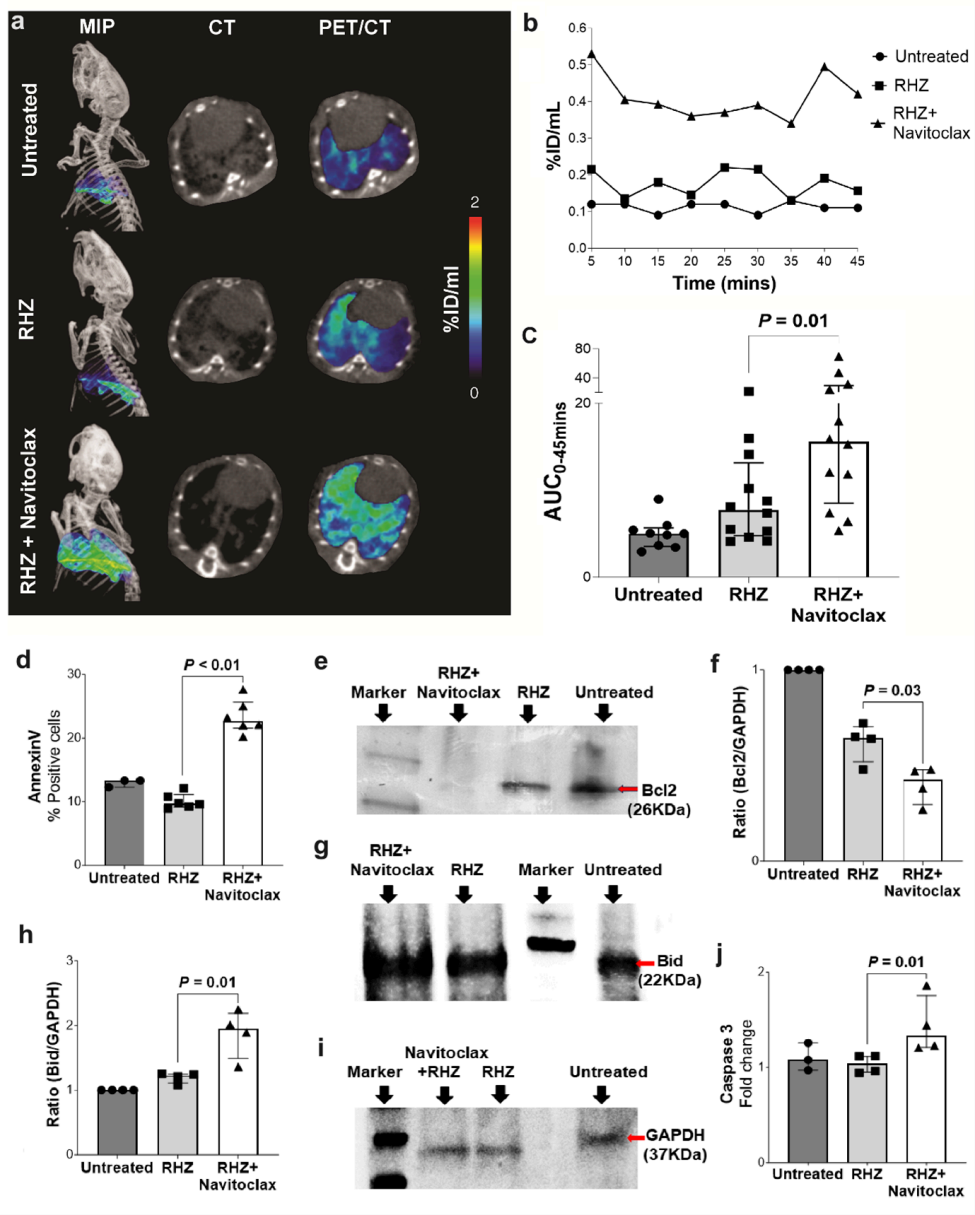
Apoptosis imaging in live *M. tuberculosis*-infected mice. **a,** Maximum intensity projection (MIP) and transverse ^18^F-ICMT-11 PET/CT from representative *M*. *tuberculosis*-infected mice from the different treatment arms, two weeks after initiation of TB treatments. Quantification of pulmonary ^18^F-ICMT-11 PET signal as percent injected dose/mL (%ID/mL) (panel **b**) and heatmaps representing area under curve (AUC) (panel **c**) (n = 3–4 mice/group) are shown. **d,** Flow cytometry of single-cell suspensions to analyse the percentage of Annexin V positive cells (n = 3 animals per group; samples were acquired in duplicates for some groups). Levels of anti-apoptotic protein Bcl2 (panels **e** and **f**), and proapoptotic protein Bid (panels **g** and **h**) from lung tissue homogenates using GAPDH as an internal control (panel **i**) are shown (n = 4 animals per group). **j**, Lung tissue homogenate caspase 3 activity is shown (n = 4 animals per group). Data are represented as median ± interquartile range. Statistical comparisons were made using the Mann-Whitney U test.

We have previously reported ^18^F-ICMT-11 PET as a non-invasive approach to measure intralesional proapoptotic responses *in situ* in mice^21^. Dynamic PET was performed in live *M. tuberculosis*-infected mice within sealed biocontainment cells^22,23^ (Fig. 2, S4). ^18^F-ICMT-11 PET area under the curve (AUC) was significantly higher in the lungs of animals treated with the standard TB treatment in addition to navitoclax versus those receiving the standard treatment alone (*P* = 0.01) (Fig. 2c). Pulmonary ^18^F-ICMT-11 PET activity was lowest in the untreated animals. To delineate the mechanistic basis of navitoclax effects, we assessed the tissue levels of anti-apoptotic protein Bcl-2, which is inhibited by navitoclax, and Bid (proapoptotic protein) in whole lung lysates using Western blots. Bcl-2 protein level was significantly lower (*P* = 0.03), and Bid level was significantly higher (*P* = 0.01) in animals receiving adjunctive navitoclax versus standard TB treatment alone (Fig. 2e–i). Similarly, apoptosis markers, Annexin V (Fig. 2d, S5), and caspase 3 (Fig. 2j) were significantly higher in mice receiving navitoclax plus standard TB treatment versus standard TB treatment alone (*P* ≤ 0.01).

### Effects of Navitoclax on Immune cells in Lung tissues

**Figure 3:**
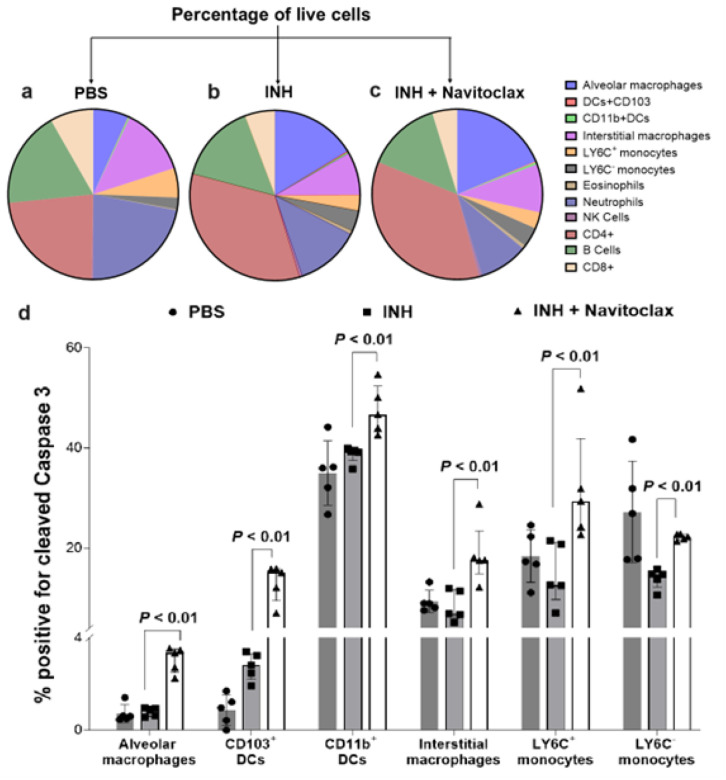
High-dimensional flow cytometry in lung tissues. Cell suspensions from lung tissues of *M*. *tuberculosis*-infected mice from the different treatment arms after exclusion of debris and doublets were analysed, two weeks after initiation of TB treatments. **a-c,** Distribution of immune cells (CD45^+^) in the different treatment arms is shown. **d,** Percentage of cells positive for intracellular expression of cleaved caspase 3 is shown. Five animals were used for each group. Data are represented as median ± interquartile range. Statistical comparisons were made using the Mann-Whitney U test.

We investigated the cell types targeted by navitoclax in the lungs of *M. tuberculosis-infected* mice using high-dimensional flow cytometry (**Fig. S6**). While the proportion of immune cells were similar in treatment groups with or without navitoclax, the addition of navitoclax led to a significant increase in apoptosis in several myeloid / macrophage lineage cells (Fig. 3; *P* < 0.01).

**Figure 4:**
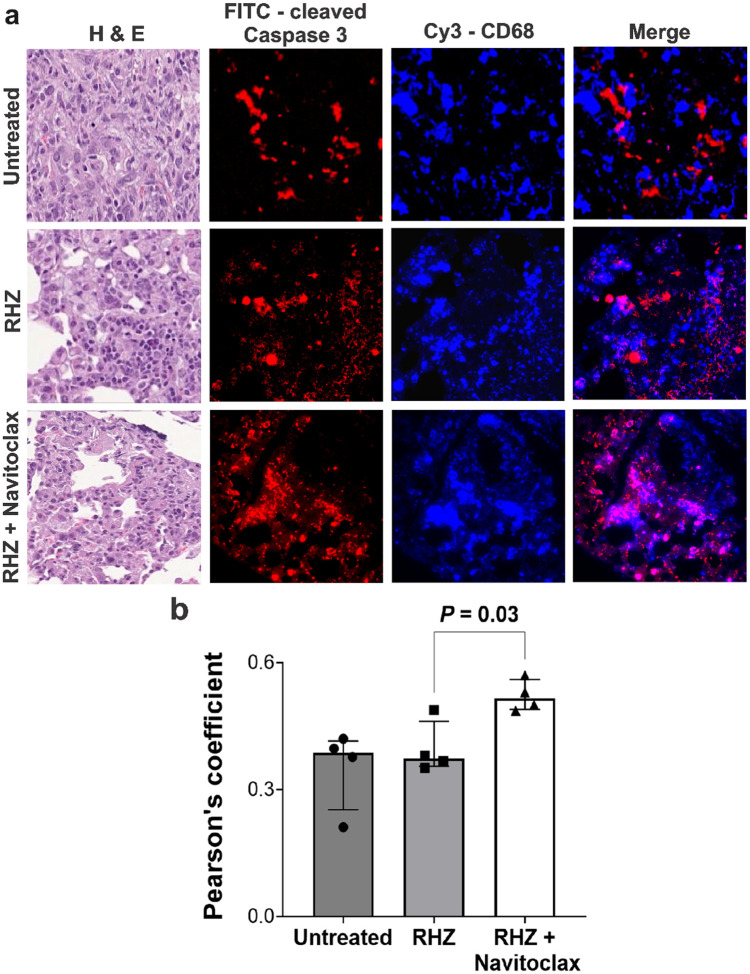
Immunohistochemistry in lung tissues. Fixed lung tissues from *M*. *tuberculosis*-infected mice from the different treatment arms, two weeks after initiation of TB treatments (n = 4 sections; 2 sections per animal). **a,** H&E-stained images and immunostained panels - cleaved caspase 3, CD68 and merged are shown (40x magnification). **b,** Pearson’s coefficient to quantify colocalization of cleaved caspase 3 and CD68 is shown. There is significantly higher colocalization of cleaved caspase 3 and CD68 in mice receiving navitoclax plus standard TB treatment versus those receiving standard treatment alone (*P* = 0.03). Data are represented as median ± interquartile range. Statistical comparisons were made using the Mann-Whitney U test.

Next, we performed immunofluorescence in lung tissue from *M. tuberculosis-infected* mice undergoing standard TB treatments, with and without navitoclax, to identify the apoptotic effects of navitoclax in key immune cells. CD68 and CD11b are markers of myeloid / phagocytic cells critical in TB pathogenesis^24–26^, and consistent with the published studies, were localized within the TB lesions in all treatment arms. Importantly, the co-localization of cleaved caspase 3 (marker of apoptosis) was significantly higher in animals receiving adjunctive navitoclax (versus standard TB treatment alone) with both CD68 (*P* = 0.03) (Fig. 4) and CD11b (*P* = 0.01) (**Fig. S7**) positive cells. The cumulative mean fluorescence intensity (MFI) for cleaved caspase 3, was significantly higher in animals receiving navitoclax plus standard TB treatment versus standard TB treatment alone (**Fig. S8**).

### Addition of Navitoclax to standard TB treatment reduces Lung fibrosis

In another set of experiments, TB treatments were initiated six weeks after an aerosol infection with *M*. *tuberculosis* (**Fig. S9**), when pulmonary fibrosis is well established in this model^27^. The overall trends in pulmonary bacterial reductions were similar to the prior experiment. However, the addition of navitoclax to the standard TB treatment reduced extrapulmonary dissemination to the spleen, with complete abrogation of brain dissemination (**Table S1**).

**Figure 5:**
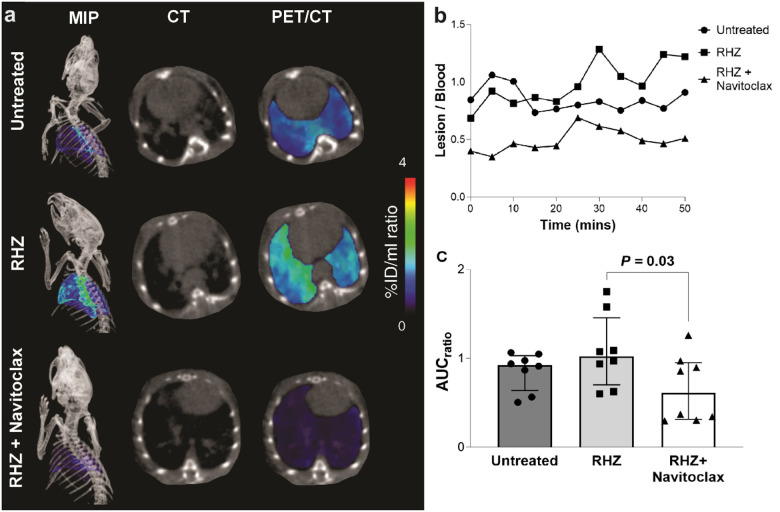
Fibrosis imaging in live *M. tuberculosis*-infected mice. **a,** Maximum intensity projection (MIP) and transverse ^18^F-FAPI-74 PET/CT from representative *M*. *tuberculosis-infected* mice from the different treatment arms, two weeks after initiation of TB treatments. Quantification of pulmonary ^18^F-FAPI-74 PET signal as lesion to blood ratio (panel **b**) and heatmaps representing area under the curve (AUC_lesion/blood_) ratio (panel **c**) (n = 4 mice/group) are shown. Data are represented as median ± interquartile range. Statistical comparisons were made using the Mann-Whitney U test.

**Figure 6:**
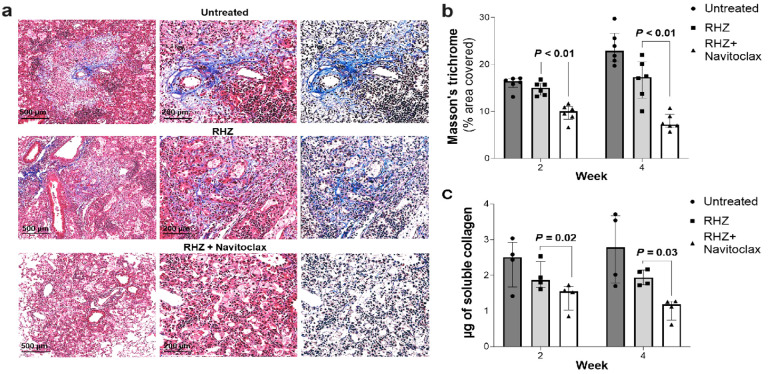
Postmortem studies to quantify lung tissue fibrosis. Fixed lung tissues from *M. tuberculosis*-infected mice from the different treatment arms, four weeks after initiation of TB treatments (n = 6 sections; 2 sections per animal). **a,** Representative Masson’s trichrome stained sections and quantification (panel **b**)**. c,** Soluble collagen was quantified in whole lung lysates (n = 3–4 animals/group). Data are represented as median ± interquartile range. Statistical comparisons were made using the Mann-Whitney U test.

Fibroblast activation protein (FAP) is a type II transmembrane serine protease, highly expressed in fibrotic tissues at the remodelling interface in lung tissues^28^. We synthesized ^18^F-FAPI-74 with a radiolabeling yield of 15.4 ± 0.1% (non-decay corrected) and a radiochemical purity of 97.5 ± 0.1%. The specific activity was 101 GBq/μmol by HPLC. (**Fig. S10**). Dynamic ^18^F-FAPI-74 PET was performed in *M*. *tuberculosis*-infected mice within sealed biocontainment cells^22,23^, demonstrating a significantly lower pulmonary PET AUC_lesion/blood_ in animals receiving adjunctive navitoclax versus standard TB treatment alone (Fig. 5, S11). The anti-fibrotic effect of navitoclax was also confirmed using postmortem studies demonstrating significantly lower pulmonary fibrosis [Masson’s trichrome staining (*P* < 0.01) and soluble collagen levels (*P* = 0.02)] in animals receiving adjunctive navitoclax versus standard TB treatment alone (Fig. 6).

## DISCUSSION

Current TB treatments comprise multidrug regimens, administered for 4–6 months, even for the treatment of uncomplicated pulmonary TB. Importantly, unlike other respiratory infections, many patients with TB have permanently damaged tissues with successful treatments only transitioning these TB patients from harboring a communicable infectious disease, to a syndrome of chronic pulmonary morbidity, commonly referred to as post-TB lung disease^29,30^. In one recent analysis of 6,225 pulmonary TB patients, abnormal lung function was noted in 46.7%, persistent respiratory symptoms in 41.0%, and radiologic abnormalities in 64.6%^30^. Although the precise mechanisms underlying post-TB lung disease remain poorly characterized, it is primarily mediated by *M*. *tuberculosis-induced* host-tissue damage (necrosis) and subsequent fibrosis^29^. Currently, there are no approved treatments to prevent post-TB lung disease. Therefore, there is significant interest in developing HDTs that can not only improve TB treatments^13–31–32^, but also maintain lung function and protect against post-TB lung disease.

During the early stages of infection, *M*. *tuberculosis* evades apoptosis via induction of anti-apoptotic Bcl-2 family proteins, leading to necrosis, increased inflammation, and vascular disruptions, ultimately leading to fibrosis^9,33^. Therefore, the strategic targeting of apoptosis using HDTs presents a novel therapeutic approach to improve TB treatments. Among the orally bioavailable, proapoptotic small molecule Bcl-2 inhibitors, navitoclax and venetoclax are available for human use, with an excellent safety profile^34^. Venetoclax is a selective Bcl-2 inhibitor and approved by the U.S. FDA^35^, while navitoclax is in clinical trials. However, we choose navitoclax for these studies as it inhibits a wide spectrum of Bcl-2 family proteins (Bcl-2, Bcl-XL, Bcl-w, Mcl-1)^36^, targets multiple host cells, including myofibroblasts, exerting anti-fibrotic effect by blocking Bcl-XL, which can treat established fibrosis in several different organs^34,37,38^, and due to its excellent safety profile. Co-administration of navitoclax with rifampin can moderately decrease navitoclax plasma levels^17^, but we demonstrate that this was not observed in our studies with *M*. *tuberculosis-infected* mice. Reversible thrombocytopenia is the only major side effect of navitoclax in human studies but daily dosing reduces thrombocytopenia risk to ~ 5%^20^, which is less than with several commonly approved antibiotics^39^. Even though daily navitoclax dosing was used in our studies, we performed platelet counts in *M. tuberculosis-infected* mice which were consistent with the reported platelet counts for untreated adult mice^40–42^, and were no different between treatment groups with and without navitoclax.

We evaluated navitoclax at human equipotent dosing (325 mg/day) in combination with the first-line, standard TB treatment (RHZ), also administered at human equipotent dosing^37,43^. C3HeB/FeJ mice were utilized as they develop human-like TB lung pathology^3,7,27,44^ and accurately predict the effectiveness of novel TB regimens that have subsequently been translated to the clinic^27,45,46^. While navitoclax did not show any antimicrobial effect on its own, when combined with the standard TB treatment, it significantly decreased the pulmonary bacterial burden and improved lung pathology. Of note, while most HDTs decrease bacterial burden only modestly (~ 1 log_10_, presumably targeting the ~ 1–2% persister population)^47,48^, even this modest decrease in bacterial burden results in a substantial decrease (~ 50%) in relapse^47,48^, with similar outcomes anticipated with navitoclax. *M*. *tuberculosis* can disseminate outside the lungs and cause extrapulmonary TB, including TB meningitis^49,50^. We observed that mice receiving adjunctive navitoclax had significantly decreased bacterial burden in the spleen and no bacterial dissemination to the brain. This is an interesting finding and is likely due to the proapoptotic effects of navitoclax, which can decrease extralesional bacterial dissemination, and highlight the potential role of navitoclax in preventing extrapulmonary dissemination and will be the subject of future investigation.

Since molecular and cellular alterations occur earlier than structural changes, molecular imaging is a powerful tool that has augmented early diagnosis, monitoring and investigation of various diseases^51^. Tomographic molecular imaging can evaluate disease processes deep within the body, noninvasively and relatively rapidly^52^. Although already critical in the management of patients with cancer, molecular imaging has similar potential for infectious diseases to provide molecular characterization of infected lesions, changes with progression or treatments, identification of patient-specific cellular and metabolic abnormalities and holistic three-dimensional visualization, which are less prone to sampling errors^53^. Here, we utilized novel, clinically translatable molecular imaging tools to noninvasively assess navitoclax-induced pulmonary apoptosis (^18^F-ICMT-11) and TB-associated fibrosis (^18^F-FAPI-74) in *live* animals, which were confirmed using postmortem studies. In the future, we anticipate that these imaging approaches could be used to noninvasively characterize post TB-lung disease as well as evaluate novel HDTs in early clinical trials.

Since navitoclax is known to affect multiple cell types, we performed flow cytometry and immunofluorescence to define the immune cell profile as well as the key immune cell types targeted by navitoclax in our studies. Although the pulmonary immune cell profiles remained similar in mice receiving standard TB treatments, with or without navitoclax, administration of navitoclax-induced apoptosis in several myeloid / macrophage-linage of immune cells. Additional studies utilizing immunofluorescence with CD11b, a pan myeloid marker and CD68, a marker for monocytes and macrophages^26,54–56^ confirmed that navitoclax-induced apoptosis in these immune cells. Importantly, we provide mechanistic data that the effects of navitoclax are mediated by a decrease in anti-apoptotic protein Bcl2 and increased expression of proapoptotic protein Bid. Overall, these data suggest that navitoclax can improve pulmonary TB treatments by enhancing bacterial clearance and reducing tissue pathology, supporting its role as an HDT for pulmonary TB.

## METHODS

All protocols were approved by the Johns Hopkins University Biosafety, Radiation Safety, and Animal Care and Use Committees (MO19M382).

### Animal infection and treatments

Six-to-seven week-old female C3HeB/FeJ (Jackson Laboratory) mice were aerosol infected with frozen titrated bacterial stocks of *M. tuberculosis* H37Rv using the Middlebrook Inhalation Exposure System (Glas-Col)^44^. Animals were housed within the ABSL-3 facility with *ad libitum* access to food and water. Five mice were sacrificed using isoflurane (Henry Schein) overdose one day of infection to assess implantation and just prior to treatment initiation to assess the bacterial burden. At the start of treatments, animals were randomly allocated to receive standard TB treatment with or without navitoclax. Untreated animals served as controls, and in some studies, navitoclax was administered alone. All drugs were administered via oral gavage, five days per week, at human equipotent dosing: rifampin (10 mg/kg/day), isoniazid (10 mg/kg/day), pyrazinamide (150 mg/kg/day) and navitoclax (100 mg/kg/day; MedChemExpress)^37^. After animal euthanasia, whole organs were removed aseptically, homogenized in phosphate-buffered saline (PBS), and plated by serial dilution onto Middlebrook 7H11 agar plates, which were incubated at 37°C for three weeks before CFU were counted.

### Imaging

Imaging was performed in live *M*. *tuberculosis*-infected mice within sealed biocontainment cells^22,23^ using the nanoScan PET/CT (Mediso). ^18^F-ICMT-11 was synthesized using an acetal protected tosylate precursor^21,57^, while ^18^F-FAPI-74 was synthesized as outlined (**Fig. S10**)^58,59^. For anatomical co-registration, a CT was acquired following the PET. Four hours after oral administration of navitoclax, mice received 3.09 ± 0.88 MBq of ^18^F-ICMT-11 via the tail vein and dynamic PET (45 min) was performed 15 min post-tracer injection. Dynamic PET (60 min) was performed immediately after an intravenous injection of 3.93 ± 0.86 MBq of ^18^F-FAPI-74 via tail vein. Volumes of interest (VOIs) were drawn manually using the CT as reference using VivoQuant 4.0 (Invicro) and the PET signal quantified from the registered images^52^. Heatmap overlays were created using AMIRA 5.2.1 (Visage Imaging, Inc.) and AMIDE 1.0.6 (Andreas Loening). ^18^F-ICMT-11 PET was represented as percent (%) injected dose (ID) per volume of tissue (mL)^21^. ^18^F-FAPI-74 PET was represented as lesion to blood AUC ratio^58,59^, with blood signal obtained by placing a VOI in the left ventricle of the heart.

### Histopathology and Immunofluorescence

Lungs were harvested after systemic perfusion with PBS, fixed in 4% paraformaldehyde. The lung lesions were identified on H&E stains and quantified using ImageJ (NIH). Masson’s trichrome stains were used to assess fibrosis and quantified using ImageJ (NIH) using Colour Deconvolution2 plugin enabled with Masson’s trichrome vector for analysis.

Immunostaining was performed at the Johns Hopkins Oncology Tissue Services Core on formalin-fixed, paraffin-embedded sections using a Ventana Discovery Ultra autostainer (Roche Diagnostics). Primary antibody (anti-CD68, 1:300 dilution, ab125212, Abcam; anti-CD11b, 1:9000 dilution, ab133357, Abcam; anti-cleaved caspase 3, 1:1000 dilution, 9661S, Cell Signalling Technology) was applied at 36°C for 40 minutes. Primary antibodies were detected using an anti-rabbit HQ detection system (7017936001 and 7017812001, Roche Diagnostics) followed by OPAL 520 (FP1487001KT, Akoya Biosciences) diluted 1:150 in 1X Plus Amplification Diluent (FP1498, Akoya Biosciences). Image acquisition was performed using the Leica DM6B system (Leica).

### Plasma and lung homogenate assays

Plasma and lung tissue homogenates were extracted and navitoclax levels were measured using mass spectrometry (Johns Hopkins Oncology core) four hours after receiving 100 mg/kg of oral navitoclax. For studies used to determine platelet levels, fresh blood was collected in EDTA tubes. Blood smears were fixed in cold methanol followed by Wright-Giemsa staining (ab245888) and counted at 100x magnification. The platelet count was obtained using methods described previously^40–42^.

Soluble collagen was quantified in whole lung homogenates using fluorometric Soluble Collagen Quantification Assay Kit (Sigma, CS0006). Fluorescent intensity was measured at 465 nm (excitation 375 nm) and μg of soluble collagen was calculated using a standard curve.

Western blot analysis was performed using a standardized protocol using primary antibodies specific to GAPDH (MA5–15738, Thermo Fisher, dilution 1:1,000), Bid (ab272880, Abcam, dilution 1:1,000), and Bcl-2 (ab182858, Abcam, dilution 1:1,000) and a goat, anti-Rabbit (ab97051, dilution 1:5,000) secondary antibody. The protein bands were visualized on the membranes using chemiluminescent substrates (Supersignal West Pico maximum sensitivity substrate, cat. no. 34580) and analysed using FIJI ImageJ.

Caspase 3 activity was quantified four hours after oral administration of navitoclax in mice using the caspase 3 assay kit (Abcam, ab39383) according to the manufacturer’s protocol. caspase 3 activity was quantified as fold-increase relative to uninfected animals.

Annexin V (Thermo Fisher Scientific, A13199) assays were performed using single-cell lung tissue suspensions analysed using the LSRII flow cytometer (BD) and Flowjo v10.8 software (BD).

### Flow cytometry

Three weeks after an aerosol challenge with *M. tuberculosis* mice were randomly allocated to receive PBS, or isoniazid with or without navitoclax. All drugs were administered via oral gavage, five days per week, at human equipotent dosing: isoniazid (10 mg/kg/day), and navitoclax (100 mg/kg/day; MedChemExpress). Two weeks after treatment initiation, mice were sacrificed with isoflurane overdose, lungs were harvested and single-cell suspensions were prepared. Surface staining was performed by incubating samples with a master mix of surface antibodies (**Table S2**). For caspase 3 staining, primary and secondary antibodies were added sequentially during permeabilization. Flow cytometry was conducted using the FACS ARIA II. The gating strategy adhered to guidelines from the American Thoracic Society (**Fig. S6**). Initial steps involved removing debris, excluding doublets and dead cells, identifying immune cells (CD45+), and excluding lymphoid cells (CD3+, CD19+, CD19). Myeloid cells were further delineated based on CD11b and CD11c positivity.

### Statistical Analysis

Data were analysed using Prism 10 Version 10.1.1 (GraphPad). Bacterial burden (CFU) are represented on a logarithmic scale (base 10) as mean ± SD and comparisons were made using a student t-test. All other data are represented as median ± IQR and comparisons were made using a Mann-Whitney U test. *P* values ≤ 0.05 were considered statistically significant.

## Data Availability

All data are available in the main text or the supplementary materials. Source data are provided with this paper.
